# Influence of Polarity of Solvents on the Spectral Properties of Bichromophoric Coumarins

**DOI:** 10.3390/molecules15128915

**Published:** 2010-12-07

**Authors:** Pavol Hrdlovic, Jana Donovalova, Henrieta Stankovicova, Anton Gaplovsky

**Affiliations:** 1 Polymer Institute, Slovak Academy of Sciences, 842 36 Bratislava, Dúbravska cesta 9, Slovakia; E-Mail: upolhrdl@savba.sk (P.H.); 2 Institute of Chemistry, Faculty of Natural Sciences, Comenius University, Mlynska dolina CH-2, SK-842 15 Bratislava, Slovakia; E-Mails: stankovh@fns.uniba.sk (H.S); gaplovsky@fns.uniba.sk (A.G.)

**Keywords:** fluorescence, bichromophoric coumarins, solvent effect, polymer matrices

## Abstract

Absorption and fluorescence spectra of bichromophoric coumarins were investigated in different solvents and in polymer matrices. These bichromophoric coumarins were composed of a coumarin dimethylamino-substituted at position 7 or unsubstituted coumarin and phthalimide or a 1,8-naphthylimide linked with an iminomethyl bridge to the position 3 or 8 of the coumarin ring. Absorption spectra of 7-dimethylamino derivatives in position 3 of coumarin were quite similar, exhibiting broad bands around 430-440 nm like the parent compound 7-dimethylaminocoumarin-3-carbaldehyde. For coumarin derivatives substituted in position 8, the absorption maximum was shifted to shorter wavelength as for derivatives without position 7 dimethylamino substitution. The most intense fluorescence was observed for 7-(*N,N*-dimethylamino)-3-[(*N*-phtalimidoyl)iminomethyl]coumarin in polar solvent, while intense fluorescence was observed for 7-(*N,N*-dimethylamino)-3-[*N*-(1,3-dioxobenz[*de*]isoquinolinyl)iminomethyl]-coumarin in non polar solvent (chloroform), comparable with the fluorescence of 7-amino-4-methylcoumarin. Spectral measurements of bichromophoric coumarins in polymer matrices revealed that the maxima lies in between those for chloroform and methanol yielding more intense fluorescence then in solutions. Completely different solvent effects were observed for 7-(*N,N*-dimethylamino)-3-[*N*-(1,3-dioxobenz[*de*]isoquinolinyl)imino-methyl]coumarin and 7-(*N,N*-dimethylamino)-3-[(*N*-phtalimidoyl)iminomethyl]coumarin. With addition of polar methanol the intensity of fluorescence decreases, yielding a Stern-Volmer-like constant of 0.54 dm^3^mol^−1^ for 7-(*N,N*-dimethylamino)-3-[*N*-(1,3-dioxo-benz[*de*]isoquinolinyl)iminomethyl]coumarin and an even higher one of 1.08 dm^3^mol^−1^ for 7-dimethylaminocoumarin-3-carbaldehyde compared to the rather low one of 0.024 dm^3^ mol^−1^ for 7-amino-4-methylcoumarin. Contrary to this, addition of methanol under identical conditions brings about an increase in fluorescence intensity of 7-(*N,N*-dimethylamino)-3-[(*N*-phtalimidoyl)iminomethyl]coumarin (about 60-fold). The reasons for these different solvent effects are discussed.

## 1. Introduction

Many natural and synthetic derivatives of coumarin (2*H*-chromen-2-one) are used in different applications in chemistry, biology, medicine and physics. The reasons for their wide range of applications are their spectral properties, mainly the intense fluorescence observed for many derivatives with appropriate substitution. These derivatives are an important components of fluorescence probes, sensors and switches, a topic which has been reviewed by de Silva [1,2]. The influence of environmental effects on the photophysics of substituted coumarins has been extensively studied with steady state and time resolved spectroscopy [3,4,5,6,7,8,9,10,11,12,13,14,15,16,17,18]. Therefore, the coumarin derivatives are widely used for monitoring the polarity and micro-viscosity of the environment in various simple, mixed or ionic solvents [3,4,5,6,7,8,9,10,11,12,13,14,15,16,17,18]. The strong solvent dependence of various coumarins was exploited for the characterization of micelles [19,20,21,22]. They respond spectrally respond to other cavities, such as for instance inclusion complexes [23,24], inside the cavities of porous materials or on its surface [25,26]. They are used for characterization of materials prepared by sol-gel processes [27,28,29] and also nanoparticles, e.g. silica or silver [30,31].

The fluorescence of coumarins is widely used as a research tool in polymer science [32,33]. Moreover, they are used as photoinitiators [34] for building up polymer chains by copolymerization [35], for estimating polymer solvent effects [36,37,38], various structural characterizations [39,40], for monitoring the releasing properties of poly(methylmethacrylate) nanospheres [41] and for polymeric fluorescent solar collectors [42]. The influence of the polymer matrix on the decay of fluorescence dialkylamino-substituted coumarins [43] and the properties of photo-responsive hyperbranched polyesters [44] have been systemically studied. Coumarins can serve as model compounds for lignin [45] and for the characterization of the polarity of cellulose surfaces [46]. Coumarins were used as a component of fluorescence probes based on intramolecular quenching which were employed as reporter of radical reaction in the thin polymer films [47,48]. Substituted coumarin-like chromphores were used as molecular rotors and fluorescence probes for biological studies [49] as well. 

In this study coumarins substituted with strong electron donating substituents which ensure well defined photophysical properties were combined (linked) with two other chromophores, namely phthalimide and 1,8-naphtylimide. Unsubstituted or alkyl-substituted phthalimide and 1,8-naphtyl-imide do not exhibit intense fluorescence and the overall solvent effect on spectral properties is not at all well pronounced. Contrary to this phthalimides substituted in position 4 with amino or dimethylamino moieties are widely used probes to test the microenvironment in solution and polymer matrices and their spectral response in solution and polymer matrices has been recently summarized [50].

1,8-Naphthylimides substituted at various positions, mainly by strong electron donating substituents, is widely used as a structural unit in simple or complex dyes employed for the construction of various fluorescence probes [1,2,51,52,53,54,55,56,57,58] due to their interesting spectral properties, namely intense fluorescence. The probes composed of 1,8-naphthalimide and sterically hindered amines have been characterized in solution and in polymer matrices [52]. 1,8-Naphtylimide-sterically hindered amine adducts have been used as polymer stabilizers and fluorescent dyes (brightener) simultaneously [53]. The 1,8-naphthalimide moiety was used as a chromophore in probe sensing anions [54]. The dyad of 1,8-naphthylimide with dansylamide has been employed as a proton induced fluorescence switch [55]. Poly(amidoamine) dendrimers functionalized with 1,8-naphthalimide units were probed as potential sensors for metal cations [56]. The strong solvatochromism of 4-methoxy- or 4-phenoxy-*N*-methyl-1,8-naphthalimide was employed for testing of ethanol-water mixtures [57,58]. Novel luminescent dyes containing 1,8-naphthalimide units have been prepared and used as well [59]. The adduct formed by combination of 1,8-naphthalimide with 2-hydroxyphenylbenzotriazole and 2,2,6,6-tetramethylpiperidine stabilizers exhibited excellent photostability [60].

**Figure 1:**
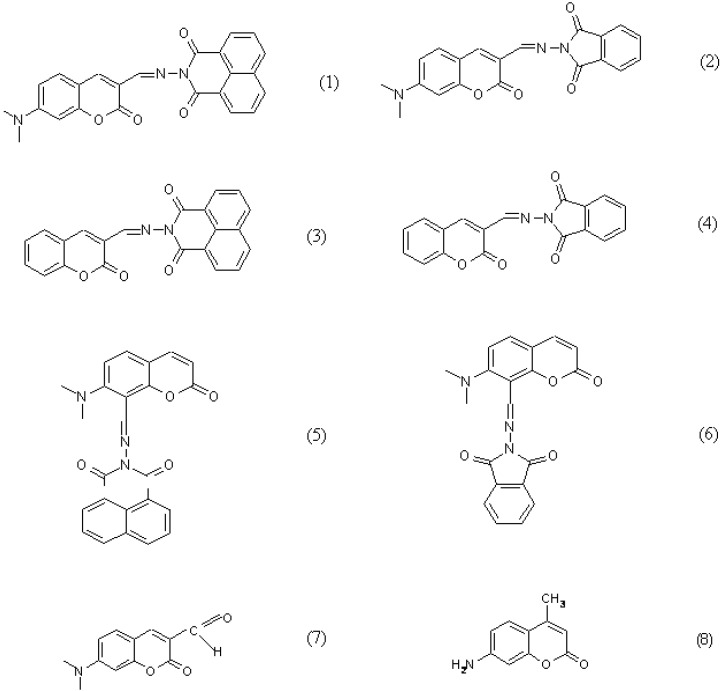
Structures of the bichromophoric coumarins and model compounds.

Strong electron donating groups such as amines, alkylamines or dialkylamines in the 4-position of the 1,8-naphthalimide skeleton causes bathochromic and bathofluoric shifts in the absorption and fluorescence spectra, respectively. Recently, we have prepared and characterized a double substituted 1,8-naphthalimide with 4-amino-2,2,6,6-tetramethylpiperidine groups in position N and 4 [61] and monosubstituted derivatives where the dimethylamino substituent was combined with a 4-amino-2,2,6,6-tetramethylpiperidine structural unit [62]. The 4-amino-2,2,6,6-tetramethylpiperidine structural unit behaves in a similar fashion as a strong electron donating group, e.g. dimethylamino.

The goal of this paper is to characterize spectrally the bichromophoric complex compound which contain as one chromophore a derivative of coumarin and as other the one phthalimide or 1,8-naphthylimide in order to gain some insight into the photophysics of these complex molecules. The spectral properties of these complex bichromophoric molecules are compared in solution and in polymer matrices. The following compounds were investigated: 7-(*N,N*-dimethylamino)-3-[*N*-(1,3-dioxobenz[*de*]isoquinolinyl)iminomethyl]coumarin (**1**), 7-(*N,N*-dimethylamino)-3-[(*N*-phtalimidoyl)iminomethyl]coumarin (**2**), 3-[*N*-(1,3-dioxobenz[*de*]isoquinolinyl)iminomethyl]coumarin (**3**), 3-[(*N*-phthalimidoyl)iminomethyl]coumarin (**4**), 7-(*N,N*-dimethylamino)-8-[*N*-(1,3-dioxobenz[*de*]isoquinolinyl)iminomethyl]coumarin (**5**), 7-(*N,N*-dimethylamino)-8-[(*N*-phthalimidoyl)iminomethyl]coumarin (**6**), 7-*N,N*-dimethylaminocoumarin-3-carbaldehyde (**7**) and 7-amino-4-methylcoumarin (**8**) (Figure 1).

## 2. Results and Discussion

The aim of the spectral study of the complex molecules which have two chromophores linked with a bridge containing double bond, was to gain some insight into their interactions. The absorption spectra of the bichromophoric coumarin – phthalimide or 1,8-naphthylimide exhibit one band in the 330 to 450 nm UV region without vibrational structure. The relevant spectral data taken in different media for series **1–8** are summarized in Table 1 and Table 2. 

**Table 1:** Absorption and fluorescence spectra of coumarins **1**-**8** in chloroform and methanol.

Substrate ^a^	chloroform					methanol			
	λ_A_^b^ nm	Logε^ c^ dm^3^mol^−1^ cm^−1^	λ_F_ nm	Φ_r_(A)^ e^	τ^f^ ns	λ_A_^ b^ nm	Logε ^c^ dm^-3^mol^−1^ cm^−1^	λ_F_^d^ nm	Φ_r_(A)^ e^
**1**	432	3.98	480	4.27	0.7	440	4.099	475	0.55
**2**	436	4.40	487	1.91	1.7	436	4.586	505	4.11
**3**	333	4.21	400	0.18		329	4.331	433	0.14
**4**	356	4.59	405	0.05		352	4.352	438	0.09
**5**	350	4.22	426	0.82	2.1	337	4.308	437	0.17
**6**	356	4.280	425	1.37	1.4	356	4.280	449	0.22
**7**	440	4.69	474	13.96	2.3	440	4.450	494	0.40
**8**	338	4.73	403	9.93	3.1	352	4.823	433	3.04

^a^ Structure of the substrates according to Figure 1; ^b^ Maximum of the longest wavelength absorption band; ^c^ Log of the decadic extinction coefficient; ^d^ Maximum of the emission band;^e^ Relative quantum yield to anthracene. Approximate absolute quantum yield is obtained by using absolute quantum yield of anthracene 0.2 for methanol and 0.11 for chloroform; ^f^ Fluorescence lifetime.

**Table 2:** Absorption and emission spectra of coumarins **1**-**8** in poly(methylmethacrylate) (PMMA), polystyrene (PS) and poly(vinylchloride) (PVC).

**Substrate^ a^**	**PMMA**				
	**λ_A_^ b^ nm**	**λ_F_^c^ nm**	**Δυ ^d^ cm^−1^**	**Φ_r_(A)^ e^**	**Τ ^f^ ns**
**1**	432	474	2 050	9.2	3.1
**2**	435	507	3 265	8.6	2.7
**3**	333				
**4**	356	432	4 994	0.8	1.4
**5**	348	405	4 404	0.53	2.0
**6**	356	418	3 398	0.72	1.7
**7**	438	477	1 866	7.57	4.0
**8**	342	399	4 177	3.53	2.6
**Substrate^ a^**	**PS**				
	**λ_A_^ b^ nm**	**λ_F_^c^ nm**	**Δυ ^d^ cm^−1^**	**Φ_r_(A)^ e^**	**Τ ^f^ ns**
**1**	433	461	1 403	3.7	1.5
**2**	432	484	2 487	3.4	1.9
**3**	334				
**4**	357	436	5 075	0.3	0.7
**5**	349	404	3 900	0.4	1.7
**6**	356	420	4 046	0.6	1.4
**7**	438	480	1 908	5.2	2.3
**8**	339	389	3 792	3.7	2.3
**Substrate^ a^**	**PVC**				
	**λ_A_^ b^ nm**	**λ_F_^c^ nm**	**Δυ^ d^ cm^−1^**	**Φ_r_(A) ^e^**	**Τ^ f^ ns**
**1**	432	486	2 520	0.4	2.6
**2**	432	510	3 540	2.5	2.8
**3**	336				
**4**	357	436	5 075	0.1	1.6
**5**	350	404	3 820	0.1	2.8
**6**	359	423	4 214	0.1	2.3
**7**	445	480	1 639	1.0	4.3
**8**	338	400	4 586	0.92	1.9

^a^ Structure of the substrates according to Figure 1; ^b^ Maximum of the longest wavelength absorption band; ^c^ Maximum of the fluorescence band; ^d^ Stoke‘s shift; ^e^ Relative quantum yield to anthracene. Approximate absolute quantum yield is obtained by using absolute quantum yield of anthracene 0.2 for PMMA, 0.16 for PS and 0.11 for PVC; ^f^ Lifetime of fluorescence.

**Figure 2:**
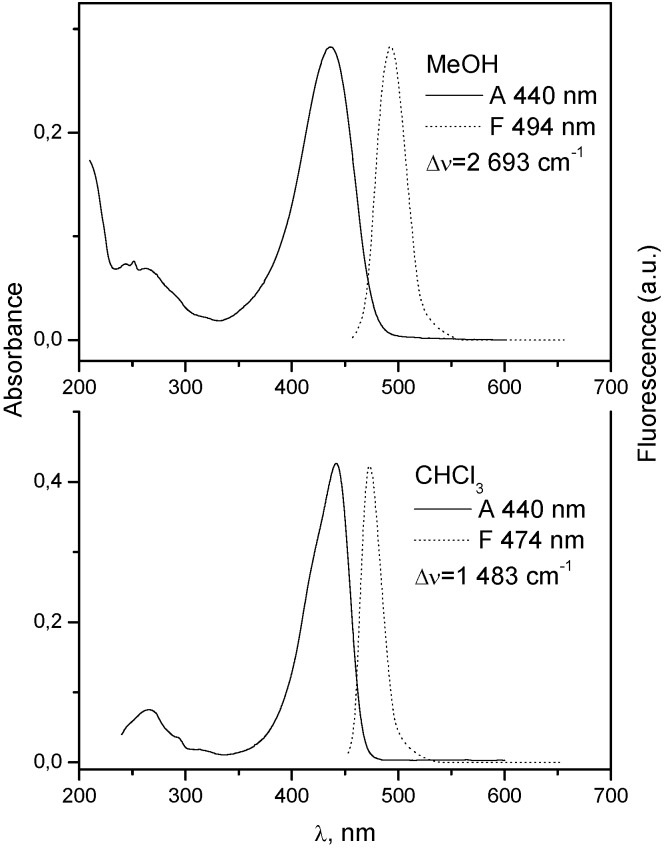
Absorption and fluorescence spectra of **7** in chloroform and methanol at 10^−5^ mol dm^−3^.

**Figure 3:**
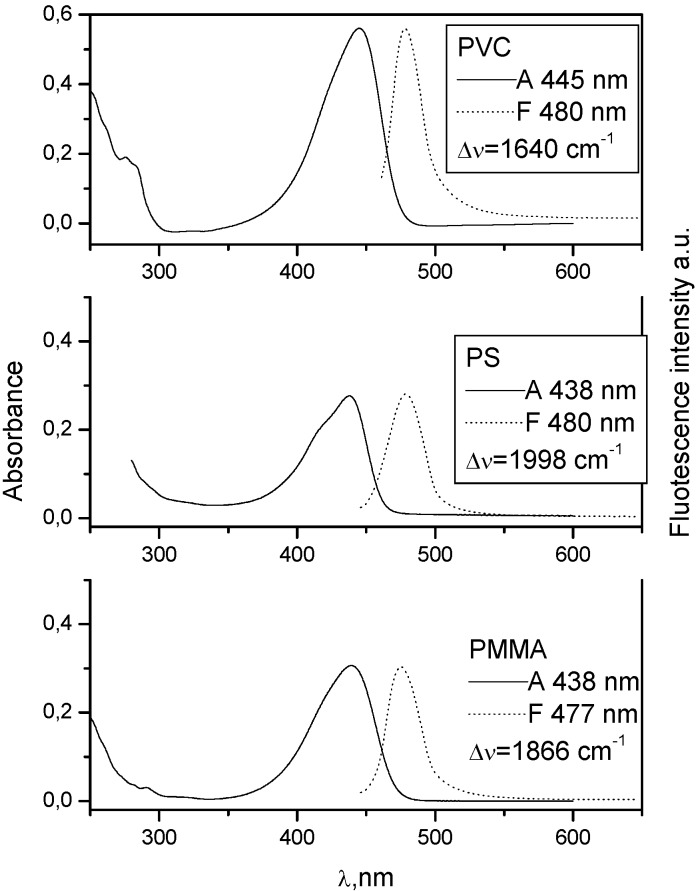
Absorption and fluorescence spectra of **7** in PMMA, PS and PVC at 0.002 mol kg^−1^.

**Figure 4:**
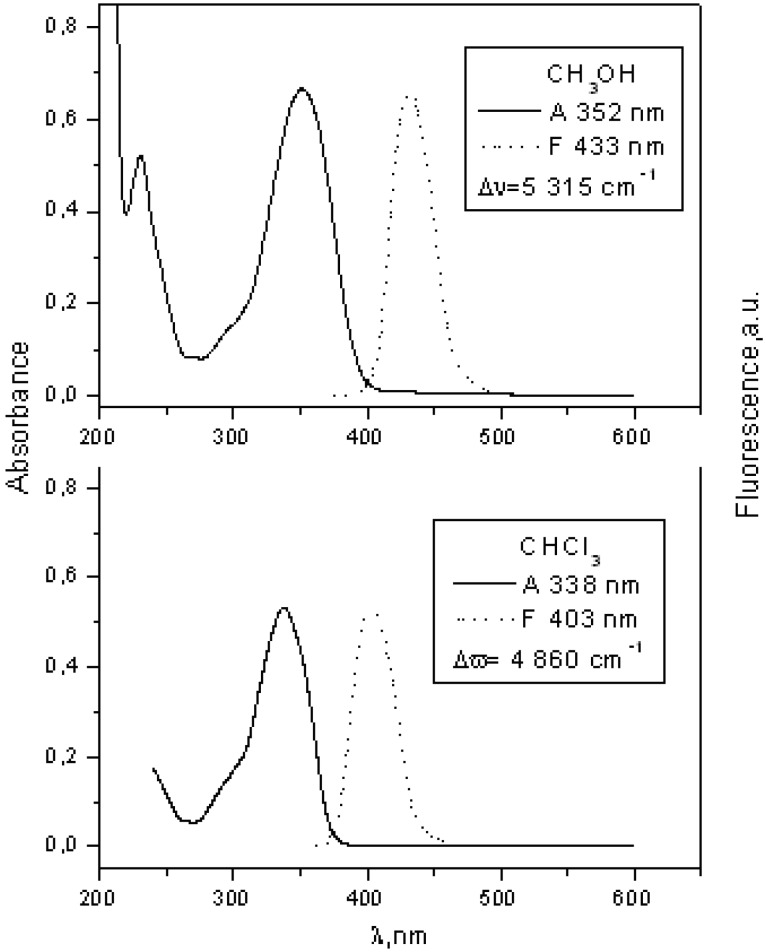
Absorption and fluorescence spectra of **8** in chloroform and methanol at 10^−5^ mol dm^−3^.

**Figure 5:**
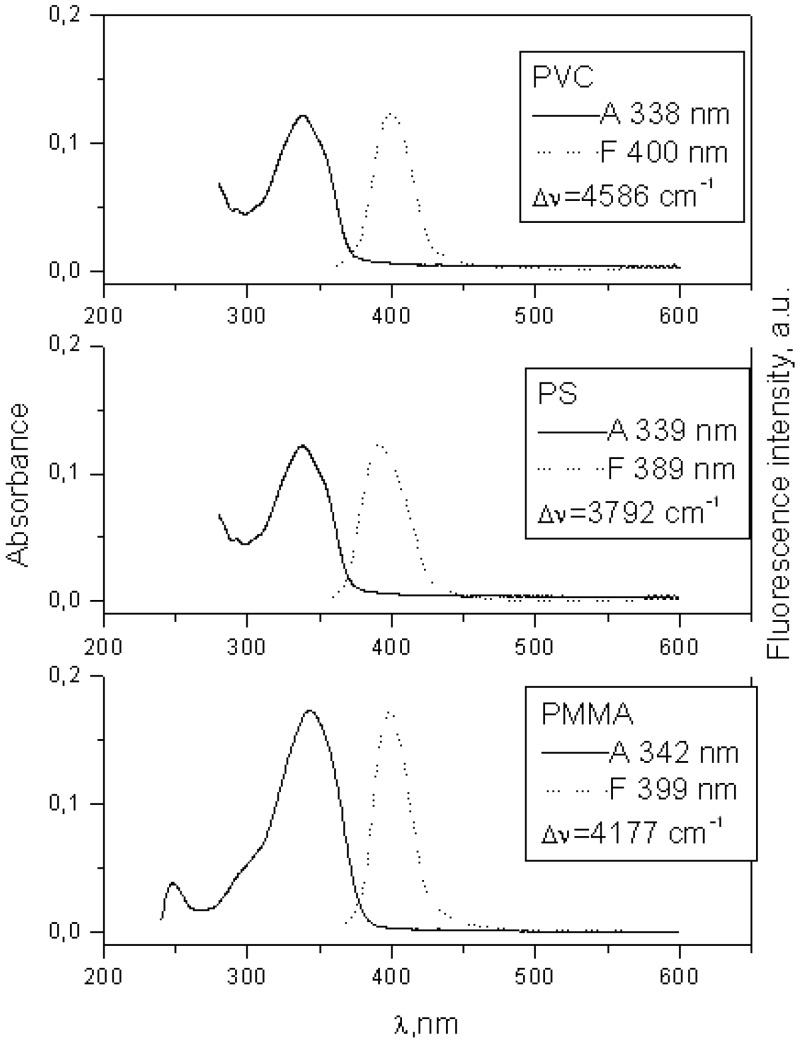
Absorption and fluorescence spectra of **8** in PMMA, PS and PVC at 0.002 mol kg^−1^.

**Figure 6:**
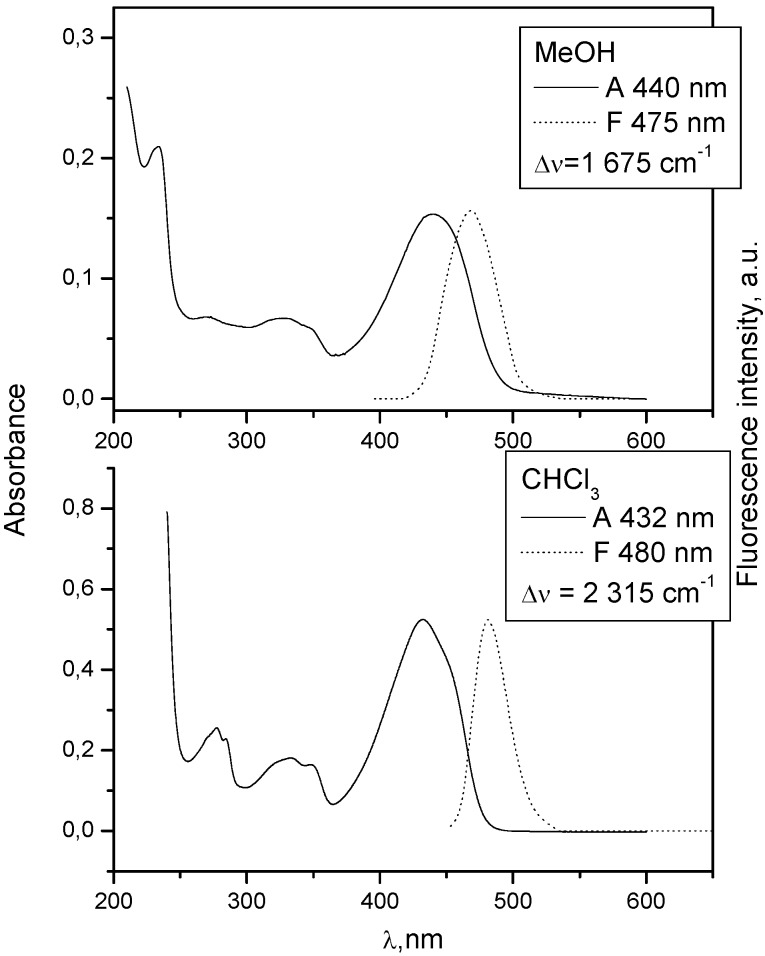
Absorption and fluorescence spectra of **1** in chloroform and methanol at 10^−5^ mol dm^−3^.

**Figure 7:**
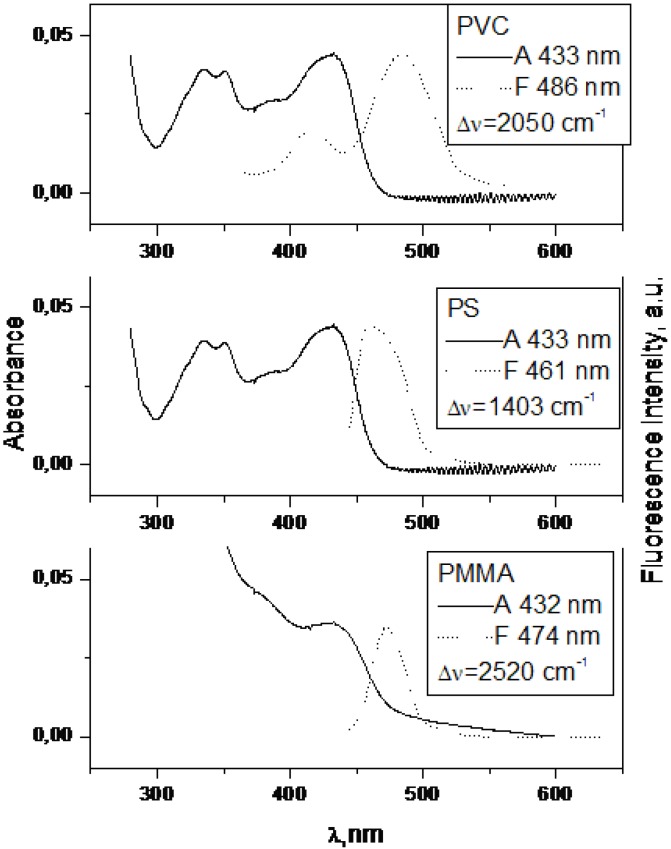
Absorption and fluorescence spectra of **1** in PMMA, PS, PVC at 0.002 mol kg^−1^.

**Figure 8:**
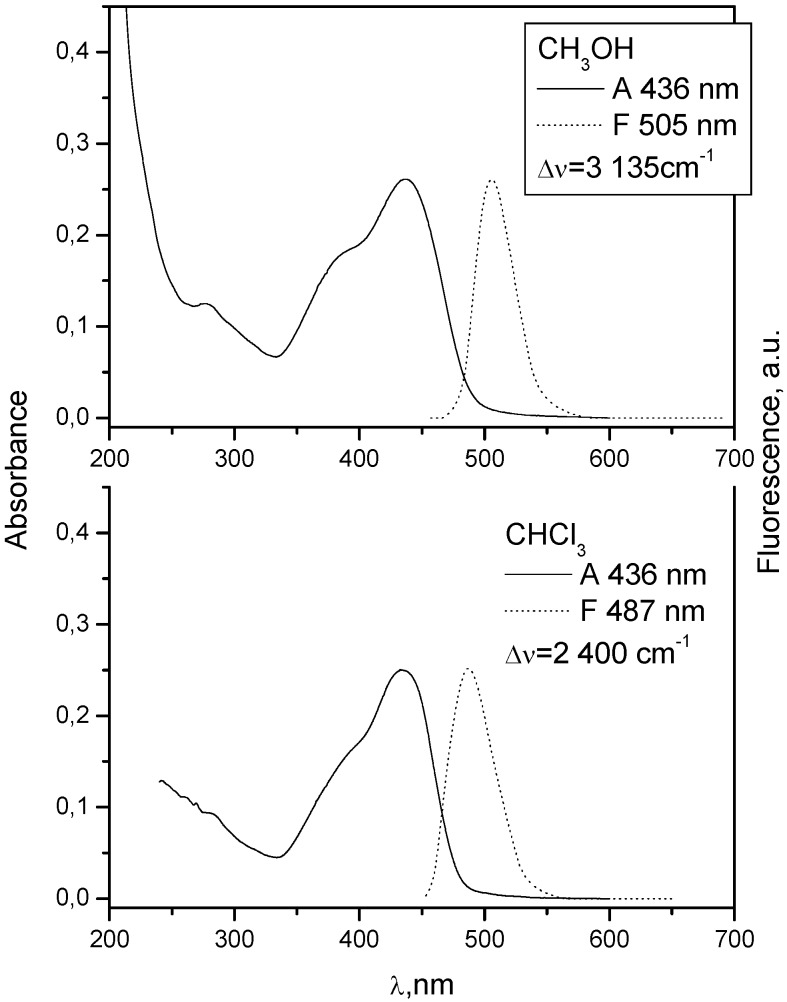
Absorption and fluorescence spectra of **2** in chloroform and methanol at 10^−5^ mol dm^−3^.

**Figure 9:**
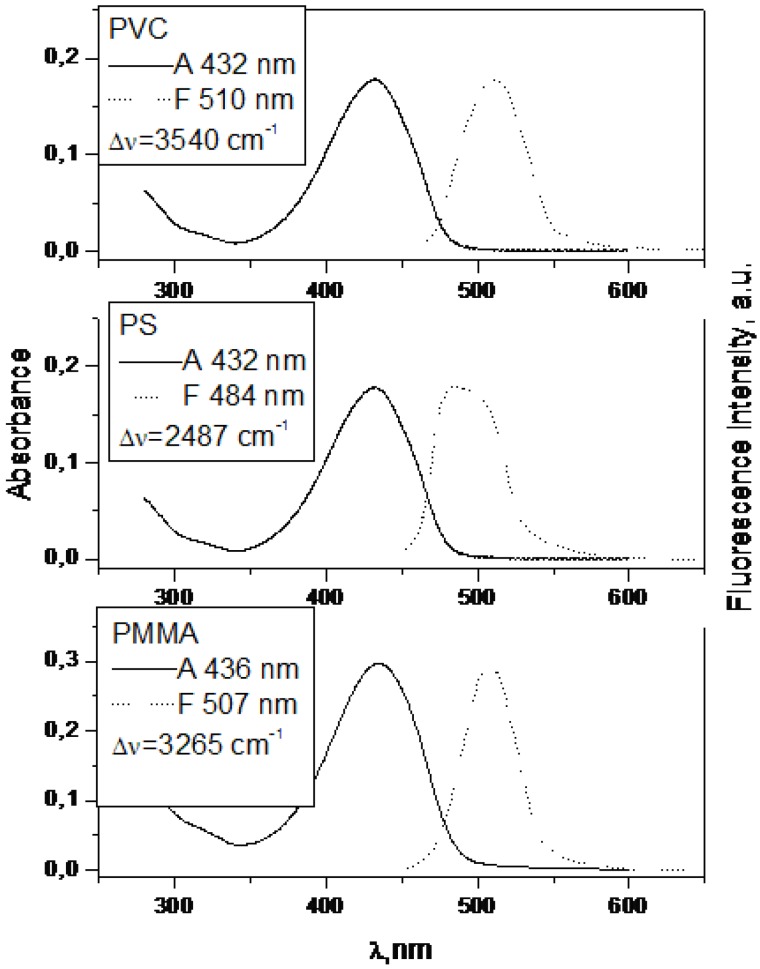
Absorption and fluorescence spectra of **2** in PMMA, PS and PVC at 0,002 mol kg^−1^.

When there is no electron donating substituent on the linked chromophores as in **3** and **4** (see Figure 1 for structures), the longest wavelength band lies at 330-350 nm. Spectra of model compounds **7** and **8** (Figure 2, Figure 3, Figure 4 and Figure 5) and bichromophoric molecules **1** (Figure 6 and Figure 7) and **2** (Figure 8 and Figure 9) indicate that the band is sensitive to substitution. 

The substitution with an electron donating dimethylamino group in position 7 shifts this band bathochromically by about 100 nm. For **5** and **6**, where the electron donating group is in position 7 on the coumarin ring and the other substituent is in position 8, the shift is shorter, about 10 nm or even less (Table 1 and Table 2) Contrary to this, model compound **8** having an amino group in position 7, taken for comparison, exhibits the longest absorption band, around 340 nm, in all examined media. The reason is the fact that there is no electron accepting group in position 3 and therefore the charge transfer interaction is rather weak in the ground as well as in the excited state. The molar decadic extinction coefficient of complex molecules and model compounds is about 10^4^ and the longest wavelength band shows a rather strong absorbance.

The fluorescence of parent bichromophoric compounds **3** and **4** is rather weak in all media (Table 1 and Table 2). Surprisingly, the derivate **3** has weak or no fluorescence, especially in polymer matrices (Table 2). The electron donating dimethylamino group in position 7 symmetrically located with respect to the aldimino group (derivatives **1** and **2**) results in increased fluorescence with a Stoke’s shift in the range 1,500-5,000 cm^−1^. The complex compounds with the same substituents but in different positions on the coumarin ring, namely 7 and 8 (**5** and **6**) exhibit less intense fluorescence around the 400-450 nm region. 

The environmental effect on fluorescence of simple or complex coumarin derivatives was extensively studied as solvent effect [3,4,5,6,7,8,9,10,11,12,13,14,15,16,17,18], inside the micelles [19,20,21,22] and cavities [23,24,25,26,27,28,29,30,31,32,33,34,35,36] and polymer matrices [32,33,34,35,36,37,38,39,40,41,42,43,44]. The fluorescence of the bichromophoric coumarins is rather sensitive on the environment. Among the bichromophoric derivatives under study only two derivatives, **1** and **2**, exhibit more intense fluorescence than anthracene and they are sensitive to the medium. The other studied derivatives like unsubstituted analogues **3** and **4** exhibit rather weak fluorescence and their medium sensitivity was not examined. Slightly more intense fluorescence is exhibited by the derivatives **5** and **6**, but no distinct medium effect was observed for them. 

**Figure 10:**
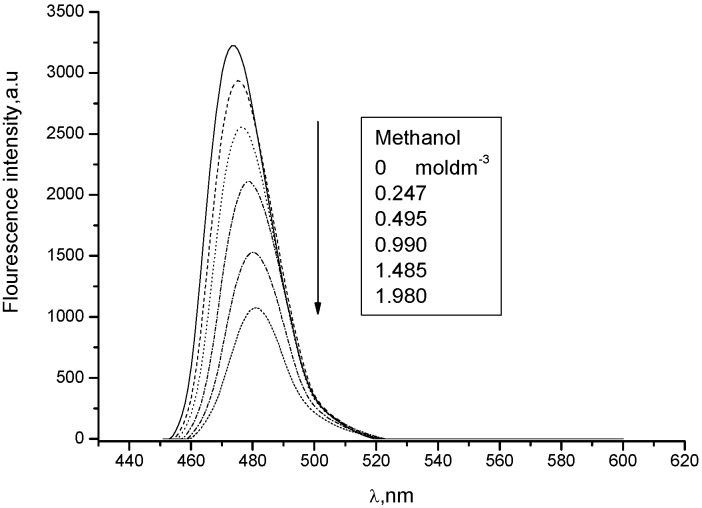
Quenching of **7** in chloroform by addition of polar methanol (0, 0.05, 0.1, 0.2, 0.3, 0.4 mL).

**Figure 11:**
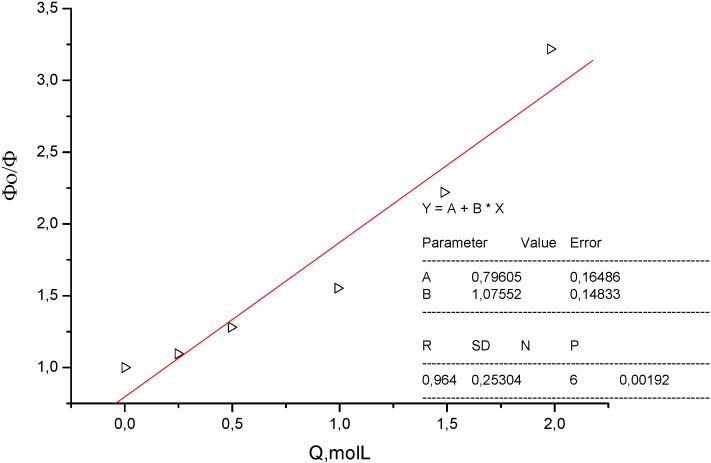
Stern-Volmer-like plot of quenching of **7** by methanol (K_SV_ = 1.08 mol^−1^ dm^3^).

The medium (solvent) effect of two substituted coumarins, which might be considered as model compounds related to bichromophoric coumarins **1** and **2** were examined for comparison as well. Since **7** exhibits considerable quenching by polar protonic solvent (K_SV_ = 1.08 dm^3^ mol^−1^) (Figure 10 and Figure 11) and there is only a small red shift (260 cm^−1^) in going from less polar chloroform to methanol, its excited state could be considered as hard anionic according to the classification of solvent effects developed by Inoue and co-workers [9].

**Figure 12:**
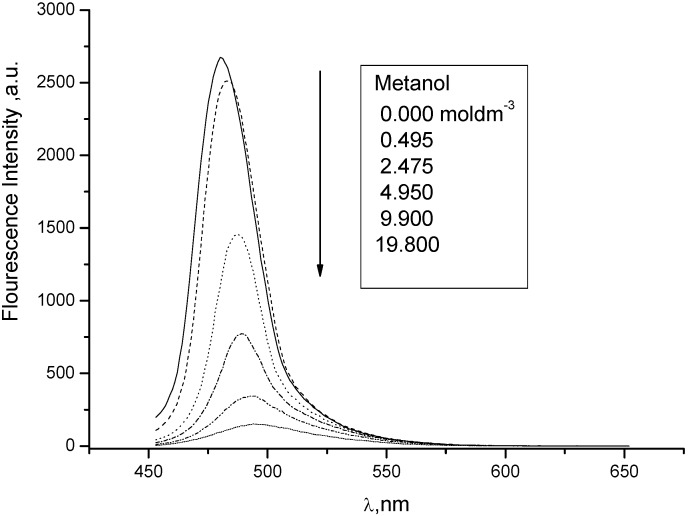
Quenching of fluorescence of **1** in chloroform by polar methanol (0, 0.1, 0.5, 1, 2, 4 mL).

**Figure 13:**
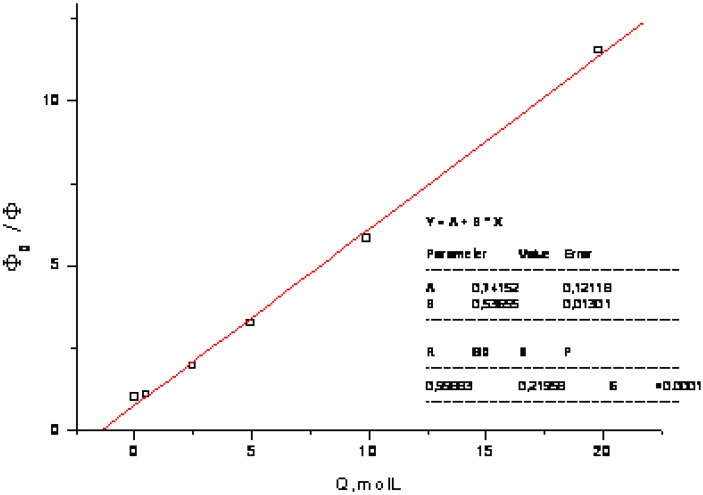
Stern-Volmer-like plot of fluorescence quenching of **1** in chloroform by polar methanol (K_SV_ = 0.54 mol^−1^ dm^3^).

A similar effect could be observed for **1** whose fluorescence is quenched by methanol slightly less effectively (K_SV_ = 0.54 dm^3^mol^−1^) (Figure 12 and Figure 13) with a larger red shift (630 cm^−1^). 

**Figure 14:**
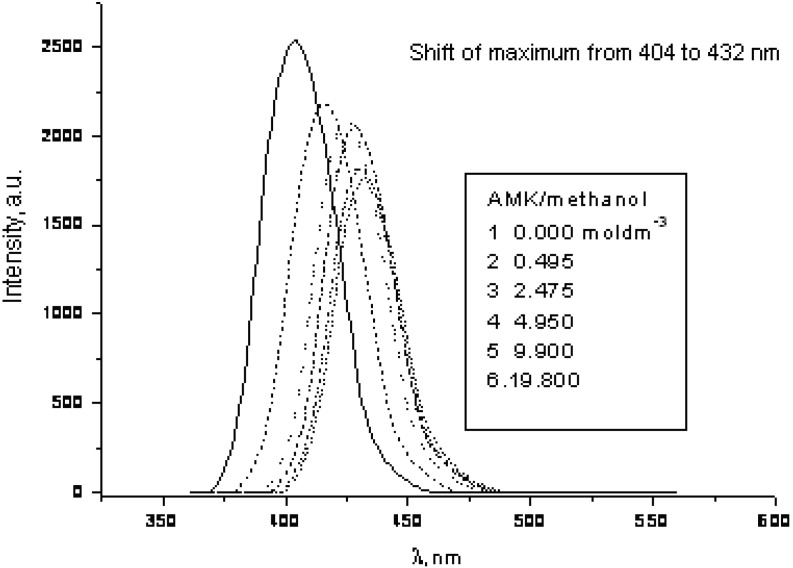
Quenching of fluorescence of **8** in chloroform by polar methanol (0, 0.1, 0.5, 1.0, 2.0, 4.0 mL).

**Figure 15:**
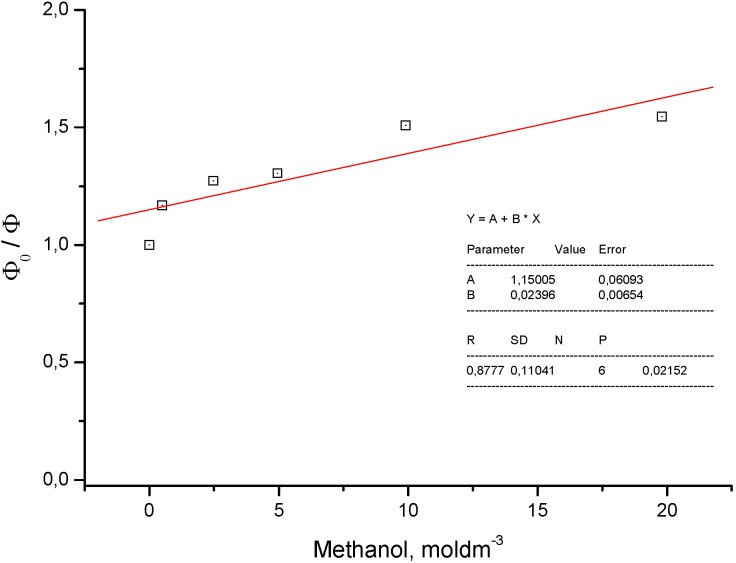
Quenching of fluorescence of **8** in chloroform by polar methanol (Stern-Volmer-like plot K_SV_ = 0.024 mol^−1^ dm^3^).

The solvent effect on the second model compound **8** involves a large red shift of about 1,600 cm^−1^ and the practical absence of quenching (0.02 dm3 mol^−1^) indicates soft anionic character of the excited state (Figure 14 and Figure 15) according to Inoue [9].

Surprisingly, **2** exhibits strong fluorescence enhancement by addition of polar methanol (up to 60-fold). The reasons for this effect are not clear. One possible explanation might be that at this compound polar protic methanol stabilizes with intermolecular hydrogen bonds the extended structure and therefore it inhibits the radiationless deactivation process and enhancement of fluorescence is observed.

## 3. Experimental

### 3.1. Synthesis of Complex Coumarins

Synthesis of bichromophoric coumarins **1-6** and parent aldehydes (Figure 1) is described elsewhere [63].

### 3.2. Spectral Measurements

Anthracene was zonnally refined (Lachema n.e., Brno, CR). Methanol was for UV spectroscopy. Chloroform, tetrahydrofuran (Slavus, SR) were analytical reagents. 7-Amino-4-methylcoumarin (**8**) used for comparison was a commercial product (Fluka, Switzerland). Polymer films doped with coumarins were prepared by casting from solution. Films of polystyrene (PS; Chemische Werke Huels, F.R.G.), poly(methyl methacrylate) (PMMA; Diacon, ICI, England) were prepared by casting of 1 mL chloroform solution of polymer (5 g/100 mL) containing the respective amount of probe on a glass plate (28 × 35 mm). The solvent was evaporated slowly. Films of poly(vinylchloride) (PVC; Neralit, Spolana Neratovice s.e., CR) were prepared by similar way by casting from tetrahydrofuran solution.

UV-VIS absorption spectra were recorded on a UV 1650PC spectrometer (Shimadzu, Japan). Emission spectra were recorded on a Perkin-Elmer MPF-4 (Perkin-Elmer, Norfolk, Conn. U.S.A.) spectrofluorimeter, which was connected through interface and A/D convertor to ISA slot of PC using a homemade data collection program. The program Origin 6.1 (Microsoft) was used for data plotting. Fluorescence of solutions were measured in a 1 cm cuvette in the right angle arrangement. The quantum yield was determined relative to anthracene in chloroform or methanol. Fluorescence of polymer films was taken in front face arrangement on the solid sample holder. The relative quantum yield of doped polymer films was determined using anthracene as standard, assuming small effect of the medium. The relative quantum yields in solution and in film were corrected on different absorption at the wavelength of excitation [64]. The fluorescence spectra were taken at excitation into the maximum of the longest wavelength absorption band.

The fluorescence lifetime measurements were performed on a LIF 200 (Lasertechnik Ltd., Berlin, F.R.G.), which operates as a stroboscope. The excitation source is a nitrogen laser emitting at 337 nm and the emission is selected by cut-off filter. The output signal of Box Car Integrator was digitized and transferred to the PC using a homemade program. The fluorescence decay curves were evaluated by simple phase plane method [65] using the program of J. Snyder based on [66]. The standard deviation G^1/2^ = Σ((I_exp_ − I_calc_)^2^/n)^1/2^, where I_exp_ and I_calc_ are intensity of emission experimental and calculated respectively, is used to judge if the decay is mono-exponential. It is assumed that the decay curve satisfies the monoexponential when G^1/2^ is lower than 5%. The fitting of fluorescence decay curves for a model of biexponential decay was preformed using adapted FluoFit MatLab package [67]. The steady state and time resolved fluorescence measurements were performed in aerated solutions. All measurements on polymer films were performed on air.

## 4. Conclusions

The spectral characterization of bichromophoric coumarins linked through an aldimino group with unsubstituted 1,8-naphthylimide and phthalimide as a second chromophore revealed that intense fluorescence is to be observed when a strong electron donating like a dimethylamino group is opposite a second chromophore (**1** and **2**). Unsubstituted (**3** and **4**) and non-symmetrically substituted derivatives (**5** and **6**) exhibited no or weak fluorescence. The derivatives exhibiting intense fluorescence exhibit opposite solvent effects, namely the fluorescence of the one with 1,8-napthylimide as second chromophore is quenched with polar, protic methanol and the fluorescence of the one with a phthalimide moiety is strongly enhanced in polar methanol. The reasons for these different solvent effects on fluorescence intensity might be due to different effect of hydrogen bond on radiationless deactivation process.
